# The Relationship between Survival and Multidrug Resistance Protein 1

**DOI:** 10.4274/TJH-2013.0150

**Published:** 2013-12-05

**Authors:** Kadir Öztürk, Yusuf EYİ, Yakup Aksoy

**Affiliations:** 1 Hakkari Military Hospital, Department of İnternal Medicine, Hakkari, Turkey; 2 Hakkari Military Hospital, Department of Emergency Medicine, Hakkari, Turkey; 3 Hakkari Military Hospital, Department of Ophthalmology, Hakkari, Turkey

## TO THE EDITOR

We read with great enthusiasm the recently published article entitled “The Frequency and Clinical Relevance of Multidrug Resistance Protein Expression in Patients with Lymphoma” by Gündüz and colleagues [1]. In that very well-designed study, they tried to evaluate the relationships between levels of multidrug resistance protein 1 (MDR1), multidrug resistance-associated protein (MRP), and lung resistance-related protein (LRP) and median survival in patients with non-Hodgkin lymphoma (NHL), Hodgkin lymphoma (HL), and reactive lymphadenopathy (LAP). They concluded that MDR1, MRP, and LRP expression did not influence overall survival in NHL and HL patients. 

The authors reported that the age difference between patient groups was not significant; the mean age was 53.2±17.5 years in the NHL patients (group 1) and 37.6±13.7 years in the HL patients (group 3), and p<0.05 was accepted as statistically significant. Although there were both statistically and clinically significant differences between the groups in age, the authors reported that there was no significant difference in age between the groups. In previous studies, it was reported that MDR1, MRP, and LRP expression levels were correlated with age [2]. In addition, in the study under present discussion, it was declared that the average survival time of patients with HL (49.50±4.69 months) was longer than that of patients with reactive LAP (42.46±5.85 months). Although the authors reported that survival of patients with HL was longer than that of the patients with reactive LAP, HL has higher mortality than in a healthy population despite all advances in treatment [3]. 

In the study, it was indicated that there was not a relationship between MDR1, MRP, and LRP expression and lactate dehydrogenase, albumin, beta-2 microglobulin, C-reactive protein, or erythrocyte sedimentation rate. However, the authors did not mention any numerical or statistical values in regard to these results. 

We thank the authors for their contribution. 

## REPLY

The mean ages for patient groups were 53.2 +/- 17.5 in non Hodgkin lymphoma (NHL) patients (group 1), 41.7 +/- 15.1 in non malignant patients and 37.6 +/- 13.7 in Hodgkin lymphoma (HL) patients (group 3). The difference was statistically significant between NHL and HL groups but there was no statistically significant difference between lymphoma patients and non malignant group. Unfortunately it was written as only “NHL patients were older than HL patients (p<0.05), the difference was not significant” with missing words. The difference was not significant sentence should be written as “the difference was not significant between lymphoma patients and non malignant group”. 

Although Schaich et al reported a correlation between age and MDR1, MRP and LRP expressions in acute myeloid leukemia patients we didn’t investigate such a relation ship. It can be analyzed in further studies. Survival of our patients with HL was 49.5+/-4.69 months. This was 42.46+/-5.85 months for NHL group. Survival was shorter than HL group for NHL patients but was not different between lymphoma groups and non malignant group. The survival is for not only reactive lymphadenopathy (LAP) but all malignant group although we mentioned as reactive LAP in our article. Non malignant group consisted of reactive LAP, granulomatous inflammation, dermatopathic LAP, benign mixed tumour and Kikuchi’s disease. These subgroups and the small number of patients in HL group might have effected our results. 

In the study MDR1, MRP and LRP expressions were not related with lactate dehydrogenase, albümin, beta 2 microglobulin, C reactive protein or erythrocyte sedimentation rate. The numerical and statistical values are not given because we didn’t want to confuse with many numbers and can be shared if requested. 

**Zafer Gülbaş**
